# The cost of being gluten-free: a hedonic pricing analysis of food products for celiac patients

**DOI:** 10.1186/s13561-025-00677-w

**Published:** 2025-10-24

**Authors:** Laia Soler, Nicolas Borzykowski

**Affiliations:** 1 University of Lausanne, Lausanne, 1015 Switzerland; 2 University of Applied Sciences Western Switzerland in Business Administration (HEG-GE), Geneva, Switzerland

**Keywords:** Gluten-free diet, Celiac disease, Hedonic pricing, I18, Q18

## Abstract

**Background:**

The gluten-free diet (GFD) has gained interest in recent years. While evidence showing that the GFD has a positive impact on everybody's health is weak, people with specific gluten-related disorders may benefit from it. As the only available treatment for celiac disease, the GDF can however not be followed without any additional cost.

**Method:**

To measure it, we collected a rich database of gluten-free and gluten-containing products on the Swiss retail market. Using a hedonic pricing method, we disentangled the price of these products and estimated the gluten-free premium.

**Results:**

We show that gluten-free products are on average 79% more expensive than regular products, which leads to an annual food-budget increase of CHF 421 (approx. the same in USD) (+ 77%) per patient.

**Conclusion:**

These results highlight the need to reflect on the social policies accompanying celiac disease in Switzerland, as there is currently no support from the disability or health insurance for these patients.

## Introduction

Popularized by show business stars, such as Miley Cyrus or Lady Gaga and athletes like Novak Djokovic for its supposed effect on well-being and sport performance, the gluten-free diet (GFD) has gained interest in recent years. Indeed, many people adopt the GFD, although they do not suffer from a diagnosed gluten-related disorders. In Germany, it is estimated that 5% of the general population follows a GFD [1] and in Australia, this rate amounts to 11% [2]. Reasons for adopting a GFD are that it may allegedly facilitate weight loss, improve general well-being or treat autism [3]. However, these claims have not been confirmed by the scientific literature so far [4, 5].

While evidence showing that the GFD has a positive impact on everybody's health is weak, people with a specific gluten-related disorders may benefit from it. The GFD is the only available treatment against celiac disease or dermatitis herpetiformis [6], auto-immune diseases that affect about 1% of the European population [1, 2]. Failing this, celiac patients will suffer from negative health consequences, including a variety of intestinal and extra-intestinal symptoms.^1^

These symptoms imply intangible costs, and avoiding them leads to direct costs. It is indeed generally acknowledged that gluten-free food is more expensive than regular food, generating extra costs for GFD adherents (see Section “Literature review”). A recent contingent valuation has estimated the total individual costs of celiac disease in Switzerland, implicitly comprising direct, indirect and intangible costs around 1′000 CHF (approx. the same in USD^2^) per year per patient [7].

The higher price of gluten-free products might be due to several factors. From a production standpoint, the dough is harder to work with than regular gluten-containing dough, and producers must take costly measures to prevent any cross-contact with gluten. This implies higher marginal costs and limited supply. On the demand side, the willingness to pay of individuals that suffer from diagnosed gluten-related disorders is high because they have no alternative and cannot eat gluten-containing products, without affecting their health. People who follow a GFD but do not suffer from diagnosed gluten-related disorders, which are often high-income people [8], might be ready to spend more to have this allegedly “healthier” diet. The combination of higher marginal costs of production and higher willingness to pay thus suggests that gluten-free products could be more expensive, other factors remaining the same.

In Switzerland, celiac patients do not benefit from the support neither of the disability insurance nor of the health insurance for the costs linked with GFD [9]. The health insurance only takes care of the medical services needed to diagnose celiac disease or to make sure the diet is correctly followed after the diagnosis through blood analyses [10] and dietician visits [11].

However, patients with celiac disease can deduct a lump sum of CHF 2′500 per year from the federal taxable income [12] if this amount exceeds 5% of the total taxable income. This means that, no deduction will be made if the yearly income is above CHF 50′000 [13]. For low-income patients, who are allowed to deduct this amount, the maximum compensation will thus only be 2′500 multiplied by the marginal tax rate. Patients with a very low income, that do not pay taxes anyway will not benefit from this deduction.

In 2003, a political motion at the federal level [9] asking for an extension of the coverage for the incremental costs of celiac disease’s diet to adults^3^ (over 20 years old) was proposed. It was suggested that the health or disability insurance should compensate for this cost increment just as the disability insurance already does for children, under the form of a lump sum. The motion was turned down by the Federal council, the Swiss federal government.

In the United Kingdom, patients with celiac disease can get gluten-free staple food for free with a prescription of their general practitioner [14]. In France, some part of the cost of gluten-free products is taken care of by the health insurance [15]. In Belgium, patients with celiac disease receive EUR 38 (USD 43) per month to compensate the cost of the gluten-free diet. In Norway, Finland, Poland, Russia and Denmark, celiac patients also receive an amount of money every month for the diet [16]. In Canada, celiac patients can deduct the effective additional cost of the diet from their taxable income. In some parts of Argentina, food is provided directly to patients [17]. However, no compensation is allocated to patients with gluten-related disorders, which are not diagnosed with celiac disease. There are also some countries that do not offer any compensation for celiac disease, such as Spain [18].

While Switzerland is less generous, compared to some of its European counterparts regarding the compensation of celiac patients, only little is known on the extra costs associated with gluten-free food. Indeed, there are only a few studies tackling this issue and isolating the impact of gluten-free on prices in the world (see Section “Literature review”). In this context, to assess the extra costs associated with gluten-free diets and to shape a possible reimbursement for Swiss patients with gluten-related disorders, we collected a rich database of gluten-free and gluten-containing products on the Swiss retail market. Using a hedonic pricing method, we disentangle the price of these products and assess the gluten-free premium. We then draw policy recommendations related to compensation for celiac patients.

The remainder of the paper is organised as follows: Section “Literature review” reviews the literature on gluten-free premium and on hedonic pricing methods. Section “Data description” describes our database, Section “Methodology” presents the methodology, Section “Results” the results and Section “Limitations and considerations for future research” discusses and Section "Conclusion" concludes.

## Literature review

Different studies have investigated the additional cost of a basket of gluten-free products compared to regular products. A summary of their results is available in Table 1.

**Table 1:** Summary of results in the literature

Study	Country	Year	Method	Type of product	Premium
Lee et al. [19]	USA	2006	Group comparison (t-test)	Market basket (breakfast cereal, milk, coffee, chicken, wine, service meals, and snacks)	240%
Stevens & Rashid, [20]	Canada	2006	Group comparison (t-test)	Bakery, cookies, baking flours and mixes, pasta, snacks, soups and sauces, cereals, meats and alternatives	242%
Singh and Whelan [21]	United Kingdom		Group comparison (t-test), ANOVA	Branded and cheapest products of 20 categories^a^	78–580%
Fry et al. [22]	United Kingdom	2015–2016	Group comparison (t-test)	Brown bread, white bread, breakfast cereals, wholegrain flour, white flour, pizza bases, wholegrain pasta, regular pasta, crackers, biscuits	159%
Capacci et al. [23]	United Kingdom	2014	Almost Ideal Demand System	Bread and cereals	90% (£10/$16.5 per week)
Missbach et al., [24]	Austria	2014–2015	Group comparison (t-test)	Flour/bake mix, bread and bakery products, pasta and cereal-based food, cereals, cookies and cakes, snacks and convenience food	75–267%
Lambert & Ficken [25]	Australia	2012	Group comparison (t-test)	Healthy food basket	76–518%
Panagiotou & Kontogianni, [26]	Greece	2015	Group comparison (Mann–Whitney U-test)	Savoury pastries, cereals, flours, pasta, processed meat products, sweets	22–334%
FACE, [27]	Spain	2017 2018	Group comparison	Basket for a 2000 kcal diet^b^	186% in 2017 (€1040/$1175 per year) 174% in 2018 (€1028/$1211 per year)
Chrysostomou et al. [28]	Cyprus	2018	Group comparison	Healthy food basket^c^	138%−154% €34–47 ($40–55) per month
Lee et al. [29]	USA	2016	Group comparison (t-test)	Market basket (breakfast cereal, milk, coffee, chicken, wine, service meals and snacks)	183%

^a^Bread loaf, bread rolls, flaked breakfast cereal, pasta, plain flour, cream crackers, sweet biscuits, fruit pies, pizza base, whole cake, stock cubes, gravy granules, barbecue sauce, brown sauce, soy sauce, frozen beef burgers, frozen sausages, frozen chicken sauce meal, fish fingers, shepherd's pie

^b^Breakfast cereals, cereal bars, biscuits, chocolate biscuits, muffins, flours, noodles, croquettes, pizza, lasagne, tarts, puff pastry, Christmas, products, beers

^c^Basket containing liquids, grain, vegetables, fruits, diary, meat, fish and eggs, fat and "residuals", such as chocolate and ice cream

In the U.K., Singh and Whelan [21] measured that gluten-free equivalents to wheat-based products were more expensive, ranging from a 78% to a 580% increase, the premium for gluten-free equivalents compared to everyday food ranged from 2 to 124%. They mention that in 2004, the Department of Health in the UK estimated the annual cost of prescriptions for gluten-free food to be GBP 520 (approx. USD 936) per patient. The latter study also highlights the limited availability of GF products [21]. Also in the U.K., Capacci et al. [23] estimated via an almost ideal demand system model that the impact of gluten-free diet on the weekly budget for food was around £10 (approx. USD 18) compared to a regular diet, underlining that the poorest had less flexibility to adjust their consumption basket. Interestingly this amount matches the yearly estimations done by the Department of Health in the UK mentioned above, twelve years prior.

In Australia, Lambert & Ficken, [25] measured the affordability of a gluten-free healthy food basket and concluded that the basket was likely not to be affordable for the poorest households, depending on its composition. To meet their nutritional needs, single men on welfare would have to spend 74% of their income. The price difference between regular and gluten-free food ranged from 76 to 518% of the regular price.

In Cyprus, Chrysostomou et al. [28] carried out a test of the affordability of the gluten-free diet for people with low income. The Cypriot Gluten-Free Healthy Food Basket represented 59% of the guaranteed minimum income for men, thereby indicating a higher risk of nonadherence among the poorest. The Cypriot Gluten-Free Healthy Food Basket was also more expensive than the Cypriot Healthy Food Basket for both women and men, respectively, by EUR 34 and 47 (USD 40 and 55) per month.

An American study [29] compared inflation-adjusted prices for gluten-free products of 2016 to those of 2006. The average additional cost decreased from 240 to 183%. However, some products increased in price over the decade, such as bread and pasta. They also analysed the evolution of the availability of gluten-free food in the different stores surveyed. In 2006, on average, only 40% of gluten-free products could be found in traditional supermarkets, while 58% were available in 2016, compared to 100% online in both periods.

Long story short, all studies find that gluten-free products are more expensive than regular products. The premium varies from 75 to 560%, depending on the country and the type of product. As shown by the literature review summarized in Table 1, surprisingly, no study strictly uses hedonic pricing to disentangle prices among products' characteristics. However, it is arguably a good tool to do so, allowing to control for all other observable characteristics, which group-comparisons are unable to do. This method thus brings more precision and reliability than simpler methods. Theoretical foundations of hedonic pricing have been developed by Lancaster [30] and formalized by Rosen [31] on the hypothesis that consumers' choices are driven by the product's characteristics rather than by the product itself. In other words, the price of the product can be explained by a series of characteristics.

Since this seminal work, the method has been extensively applied by environmental economists using housing markets data.^4^ However, the power of the method is that it can be used on any differentiated product, with any market data, including the food market. Furthermore, by including all types of products in a single regression, it keeps statistical power to a maximum. A review of hedonic analysis using food markets can be found in Costanigro & McCluskey [32].

In what follows, we applied the hedonic pricing method to assess the price difference of gluten-free products vs. regular products, to draw policy recommendations, design compensation for celiac patients and open avenues for further research.

## Data description

In the light of the previous results, the extra cost of gluten-free products in Switzerland was assessed via a hedonic pricing method on a new rich database of food products, collected in 2019. The data collected for this study are from the websites of the two main Swiss supermarkets chains: leshop.ch and coopathome.ch because these are the most common places to buy groceries– Coop and Migros together represent close to 70% of the market share in the food industry [33] – and they were the only two supermarkets offering food products online.

The extra costs of GFD was estimated thanks to this database of prices and then compared with 1) the Swiss household budget survey led by the Swiss Federal Statistical Office [34] and 2) the estimations of celiac patients in a questionnaire passed among members of the Swiss French-speaking celiac disease association (see Soler [35] for more information). The latter questionnaire aimed at assessing the different types of costs (direct, indirect, and intangible) supported by celiac patients and allowed assessing the extra costs of GFD, from the perspective of celiac patients. The latter specific estimation is important since celiac patients' consumption patterns might be different from the regular population.

Comparing the estimated GF premium with other data sources allowed assessing the total cost of the GFD in the total household's budget.

The final database is based on 552 products, of which 126 are gluten-free products. The products were divided into 15 categories according to the type of food, such as pasta, toast bread, or pizza. Each observation also includes information on whether the product is organic, using wholemeal flours, is of a budget brand or a premium brand. Products whose price would be affected by other determinants (for example sugar-free, low fat or fitness products) were excluded from the database.

The selection of products was made as exhaustive and consistent as possible. Since the goal is to have the most meaningful comparison between the gluten-containing and gluten-free products, either one very specific type of product was chosen (chocolate chip cookies, *Walkers* and wafers instead of all the different types of biscuits, because only few exist in the gluten-free offer) or all the varieties were included, as they were considered all equivalents (for pasta for example). Prices have been standardised to a 100 g or 100 ml of product.

**Fig. 1:**
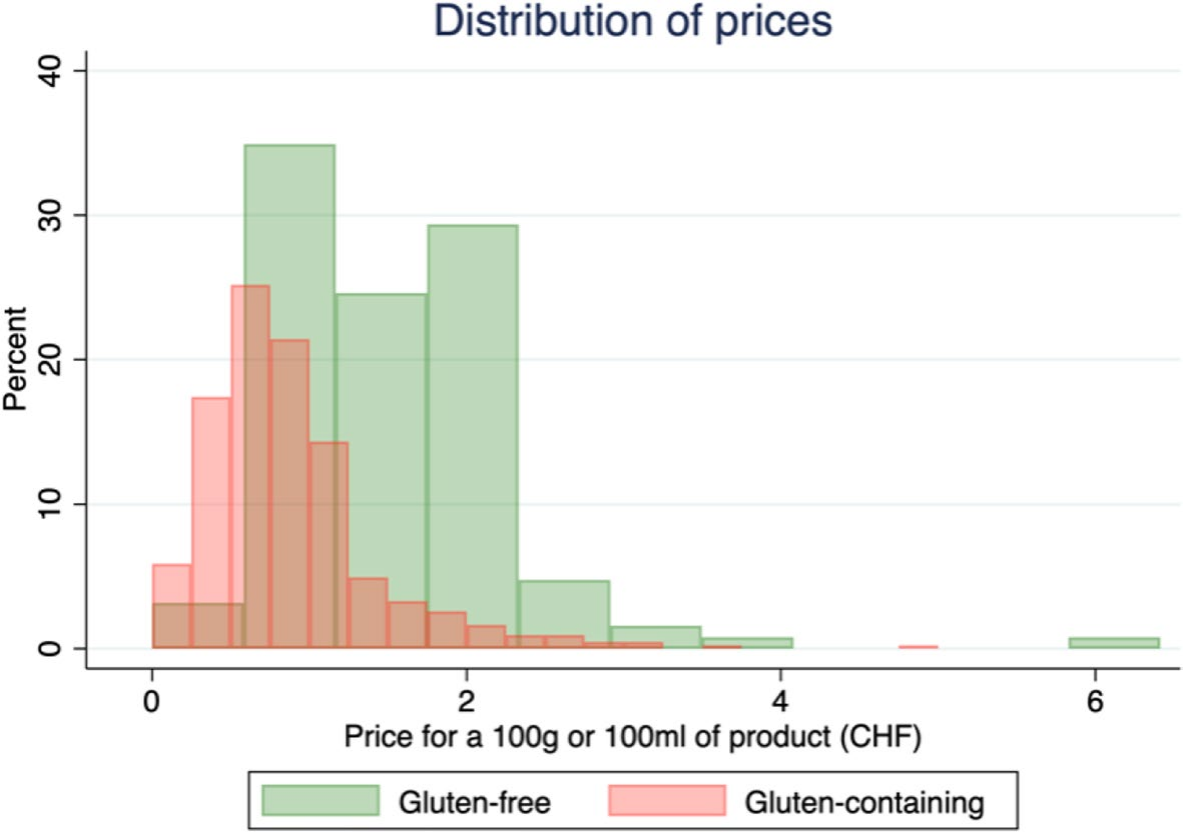
Distribution of prices per 100 g of food. This graph represents the distribution of prices per 100 g of food (or 100 ml for liquids) for gluten-containing (regular) products (*n* = 426) and for gluten-free products (*n* = 126)

**Fig. 2:**
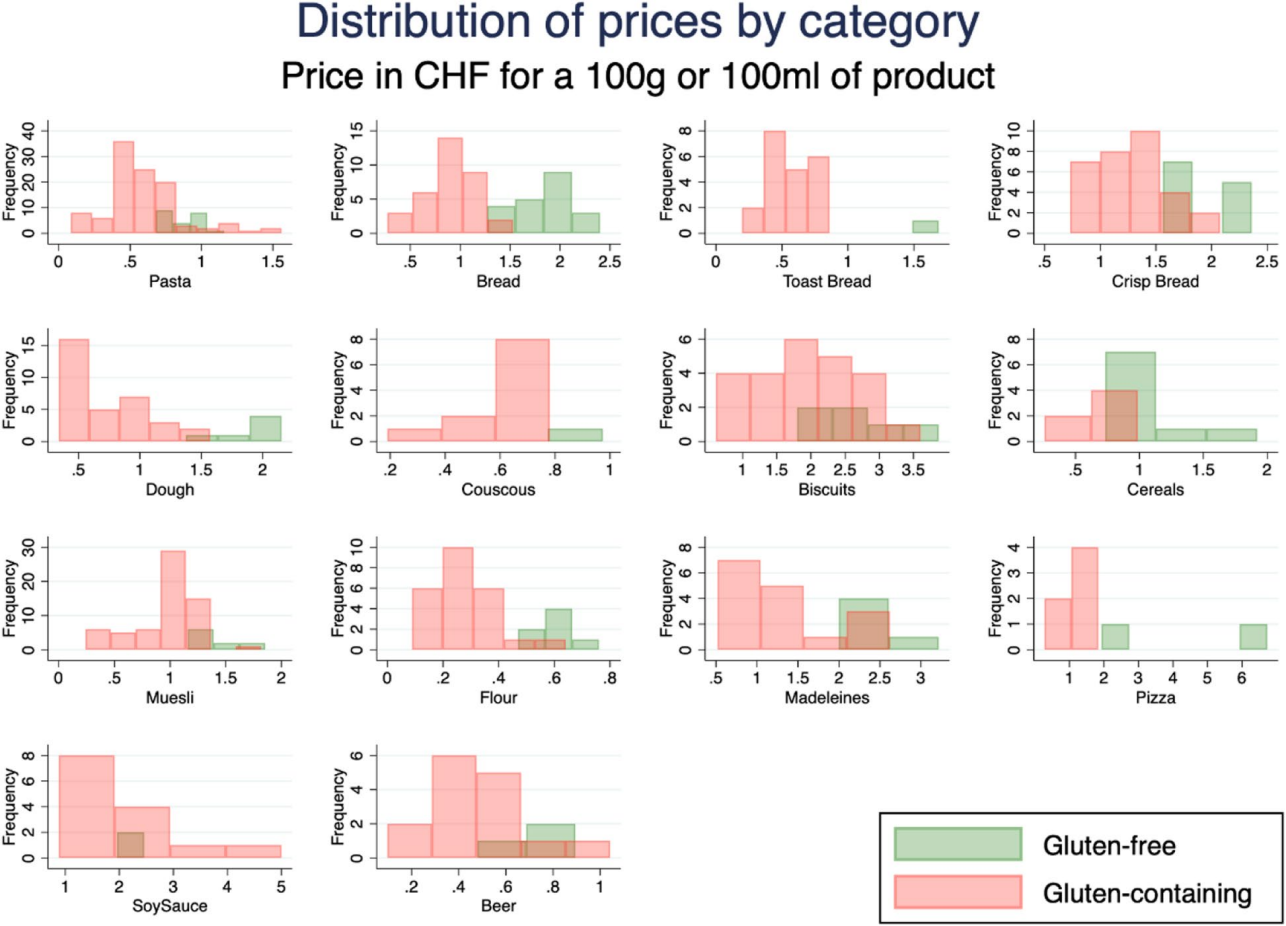
Distribution of prices by category of products. These different graphs show the distribution for 100 g/100 ml of product for each category separating gluten-containing (regular) and gluten-free foods

Figure 1 suggests that, overall, gluten-free products are more expensive than gluten-containing products. The distribution of the prices is skewed to the right. However, the shape of the distribution for each category does not have a common pattern (Fig. 2). This figure also shows that, in general, there are fewer gluten-free products available, and they seem to be more expensive for most of the categories.

Table 2 compares the price in each category for the gluten-free and regular products. The cheapest gluten-free products are three times and up to nine times the price of the cheapest regular products. The price difference for gluten-free products implies that for the smaller budgets, adopting a gluten-free diet is more difficult.

**Table 2:** Descriptive statistics and comparison of gluten-free (GF) vs gluten-containing (GC) products

	**Number of observations**			Mean price CHF/100 g*		**Minimum price CHF/100 g**	
**Category**	**Total**	**GF**	**GC**	**GF**	**GC**	**GF**	**GC**
Pasta	129	22	107	**0.86 (0.14)**	**0.58 (0.27)**	0.68	0.09
Bread	40	19	21	**1.43 (0.36)**	**0.68 (0.20)**	0.93	0.36
Toast Bread	22	1	21	1.49 (.)	0.56 (0.19)	1.49	0.20
Prebacked Bread	55	21	34	**1.81 (0.32)**	**0.91 (0.29)**	1.27	0.27
Crisp Bread	43	12	31	**1.88 (0.33)**	**1.27 (0.34)**	1.56	0.73
Dough	39	6	33	**1.85 (0.26)**	**0.72 (0.32)**	1.38	0.34
Couscous	12	1	11	0.77 (.)	0.59 (0.16)	0.77	0.19
Biscuits	30	6	24	**2.62 (0.83)**	**1.95 (0.75)**	1.80	0.61
Cereals	15	9	6	**1.06 (0.39)**	**0.66 (0.32)**	0.73	0.26
Muesli	72	10	62	**1.37 (0.23)**	**0.95 (0.29)**	1.16	0.24
Flour	31	7	24	**0.61 (0.09)**	**0.28 (0.12)**	0.47	0.09
Madeleines	21	5	16	**2.51 (0.45)**	**1.32 (0.66)**	2.00	0.52
Pizza	8	2	6	**4.17 (3.17)**	**1.25 (0.56)**	1.93	0.28
Soy sauce	16	2	14	2.00 (0.06)	2.01 (1.01)	1.96	0.88
Beer	18	3	15	0.69 (0.21)	0.45 (0.24)	0.48	0.10
Wholemeal	103	23	80	**1.53 (0.45)**	**0.72 (0.32)**		
Organic	137	37	100	**1.45 (0.64)**	**0.81 (0.43)**		
Budget	32	0	32		0.33 (0.31)		
Premium	44	7	37	0.98 (0)	1.03 (0.81)		

^*^Standard deviation in parentheses, statistically different at 10% using a t-test in bold

## Methodology

Two questions arise from this database: first whether there is a premium for gluten-free products, independently of other characteristics, and what is the amount of this mark-up. And second, how does it affect the monthly budget for celiac patients. To answer these questions, we test two models described in Eqs. (1) and (2) below.

1 $$\begin{aligned}price100g_i&=\alpha+\sum\nolimits_{c=1}^C\beta_c\times Category_{c,i}+\sum\nolimits_{c=1}^C\gamma\times Category_{c,i}\times Glutenfree_i\\&\quad+\delta_1 \times Organic_i + \delta_2\times Wholemeal_i+\delta_3\times Premuim_i+\delta_4\times Budget_i+u_i\end{aligned}$$

2 $$\begin{aligned}{\text{ln}\;(price100g}_i)=\alpha+\sum\nolimits_{c=1}^C\beta_c\times{Category}_{c,i}+\sum\nolimits_{c=1}^C\gamma_c\times{Category}_{c,i}\times{Glutenfree}_i+\delta_1\\\quad\times{Organic}_i+\delta_2\times{Wholemeal}_i+\delta_3\times{Premium}_i+\delta_4\times{Budget}_i+u_i\end{aligned}$$

The first model (Eq. 1) is linear and estimates the price of the products as follows:

- $\alpha$ represents the constant term and, in this case, it is the average price of the cheapest category of products with gluten, namely flour;
- There is a dummy variable for each category of products (*Category*_*c*_). The ${\beta }_{c}$ coefficients capture the average price increase for each category of products compared to the average of the cheapest category of products already captured by $\alpha$;
- *Gluten-free* is a dummy variable, 1 for gluten-free products, 0 for gluten-containing products. When it is multiplied by the dummy variable for the category of products, the interaction term results in a dummy variable equal to 1 for the gluten-free items of a certain category. ${\gamma }_{c}$ coefficients capture the average absolute mark-up for gluten-free products for each category;
- *Organic*, *Wholemeal*, *Premium* and *Budget* are four dummy variables that are equal to 1 when the product has the characteristic. *Organic* refers to products which have been produced without pesticide. *Wholemeal* is equal to one if the products are made with wholemeal flours. *Premium* takes the value 1 for products that are of a higher-quality brand and *Budget* includes products which are from a “low budget” brand. $\delta$ coefficients capture the effect on prices of those four characteristics. They give the absolute average effect for the whole sample (independently of category).
- The last element (*u*_*i*_) represents the error term

The second model (Eq. 2) is a log-linear model. The interpretation of this model is the following: a unit variation in an independent variable will imply a variation of the log(price) captured by the coefficient and this difference is close to the percentage variation^5^ of the actual price implied by this unit variation of the independent variable.

We also tested a Box-Cox transformation on the dependent variable. Results are similar in magnitude and signs. Therefore, we do not comment further the latter model but only provide its results in Table 7 in the Appendix.

The information about the mark-up for gluten-free products is then used to calculate the implied impact of a gluten-free diet on the monthly budget of a Swiss average person based on the Swiss households budget survey of the Federal Office of Statistics.

## Results

Both models were run using Ordinary Least Squares and robust standard errors. All the coefficients are reported in Table 3.

**Table 3:** Model 1 and 2 regression results

Model	(1) Price per 100 g		(2) LogPrice per 100 g	
Organic	0.041	(0.030)	0.084^***^	(0.029)
Wholemeal	0.077^**^	(0.032)	0.159^***^	(0.034)
Premium	0.419^***^	(0.093)	0.496^***^	(0.056)
Budget	−0.548^***^	(0.057)	−1.091^***^	(0.071)
Pasta	0.198^***^	(0.043)	0.586^***^	(0.068)
Bread	0.327^***^	(0.056)	0.812^***^	(0.083)
Toasts	0.313^***^	(0.046)	0.781^***^	(0.075)
Pre-cooked bread	0.642^***^	(0.050)	1.237^***^	(0.068)
Crisp bread	0.948^***^	(0.061)	1.515^***^	(0.073)
Dough	0.544^***^	(0.065)	1.158^***^	(0.096)
Couscous	0.305^***^	(0.058)	0.779^***^	(0.127)
Biscuits	1.755^***^	(0.145)	2.122^***^	(0.099)
Cereals	0.576^***^	(0.053)	1.208^***^	(0.075)
Muesli	0.708^***^	(0.045)	1.335^***^	(0.069)
Muffins	1.127^***^	(0.153)	1.709^***^	(0.108)
Pizza	1.073^***^	(0.153)	1.647^***^	(0.126)
Soy sauce	1.711^***^	(0.244)	1.958^***^	(0.110)
Beer	0.221^***^	(0.067)	0.527^***^	(0.133)
GF pasta	0.254^***^	(0.039)	0.472^***^	(0.048)
GF bread	0.782^***^	(0.096)	0.795^***^	(0.090)
GF toasts	0.834^***^	(0.038)	0.860^***^	(0.055)
GF pre-cooked bread	0.867^***^	(0.082)	0.660^***^	(0.060)
GF crisp bread	0.647^***^	(0.107)	0.454^***^	(0.068)
GF dough	1.040^***^	(0.116)	0.849^***^	(0.097)
GF couscous	0.164^***^	(0.051)	0.283^**^	(0.113)
GF biscuits	0.592^*^	(0.345)	0.176	(0.138)
GF cereals	0.204	(0.136)	0.171	(0.126)
GF muesli	0.380^***^	(0.076)	0.330^***^	(0.059)
GF flour	0.309^***^	(0.037)	0.824^***^	(0.065)
GF Muffins	1.116^***^	(0.240)	0.599^***^	(0.116)
GF Pizza	2.835^*^	(1.642)	1.013^**^	(0.452)
GF soy sauce	0.026	(0.240)	0.137	(0.092)
GF beer	0.204^*^	(0.116)	0.469^**^	(0.193)
Constant (Reg. Flour)	0.263^***^	(0.038)	−1.403^***^	(0.062)
Observations	551		551	
Adjusted R2	0.719		0.806	

Heteroskedasticity-robust standard errors reported in parenthesis

^*^p <.1, ^**^p <.05, ^***^p <.01

All categories of products, except for soy sauce and cereals, are significantly more expensive in their gluten-free version. The coefficients for the interaction terms that capture the effect of being gluten-free for each category (see the second half of Table 3) are highly significant for most of the categories (9 categories have a *p*-value < 0.01). The adjusted R-squared is high (0.7–0.8), implying that the prediction of the price by the models is good.

The absolute and relative increases calculated by the two models are reported in Table 4. The increase goes up to 212% for the pizza category in the linear model, and up to 175% in the log-linear model. On average, the increase in price amounts to CHF 0.68 per 100 g or 100 ml of food, which is equivalent to an average increase of 79%. Gluten-free bread is on average CHF 0.78 more expensive than regular bread, which corresponds to an increase of 131% (model 1). Gluten-free flour is CHF 0.31 more expensive, increasing by 117% (model 1).

**Table 4:** Price difference between gluten-containing and gluten-free products based on the regressions from Table 3

	**Model 1** **CHF/100 g increase**	**Model 1** **% increase**^a^	**Model 2** **% increase**^b^
GF pasta	0.254	55%	60%
GF bread	0.782	133%	121%
GF toasts	0.834	145%	136%
GF pre-cooked bread	0.867	96%	93%
GF crisp bread	0.647	53%	57%
GF dough	1.040	129%	134%
GF couscous	0.164	29%	33%
GF biscuits	0.592	29%	*19%*
GF cereals*	*0.204*	*24%*	*19%*
GF muesli	0.380	39%	39%
GF flour	0.309	117%	128%
GF Muffins	1.116	80%	82%
GF Pizza	2.835	212%	175%
GF soy sauce*	*0.026*	*1%*	*15%*
GF beer	0.204	42%	60%
**Average**	**0.684**	**79%**	**78%**
**According to celiac patients**		**223%**	

^*^In italic, results that were not significant at 10% level

^a^Percentages are drawn from comparing the predicted value of the model for gluten-containing and gluten-free products for each category setting all other control variables to zero

^b^Percentages are calculated from the log-linear coefficients

To calculate the budget impact of GFD, we need to match the categories made by the Federal Statistical Office (FSO). For this, all kinds of bread were combined into a new variable (their results were similar enough to do so) and regular flour was separated from the wholemeal flour.

Combining the FSO average household basket from the household budget survey and our results, we find that a gluten-free diet is 77% more expensive adding CHF 421 per household to the average Swiss food budget per year (see Table 6 in the Appendix for more details).

**Fig. 3:**
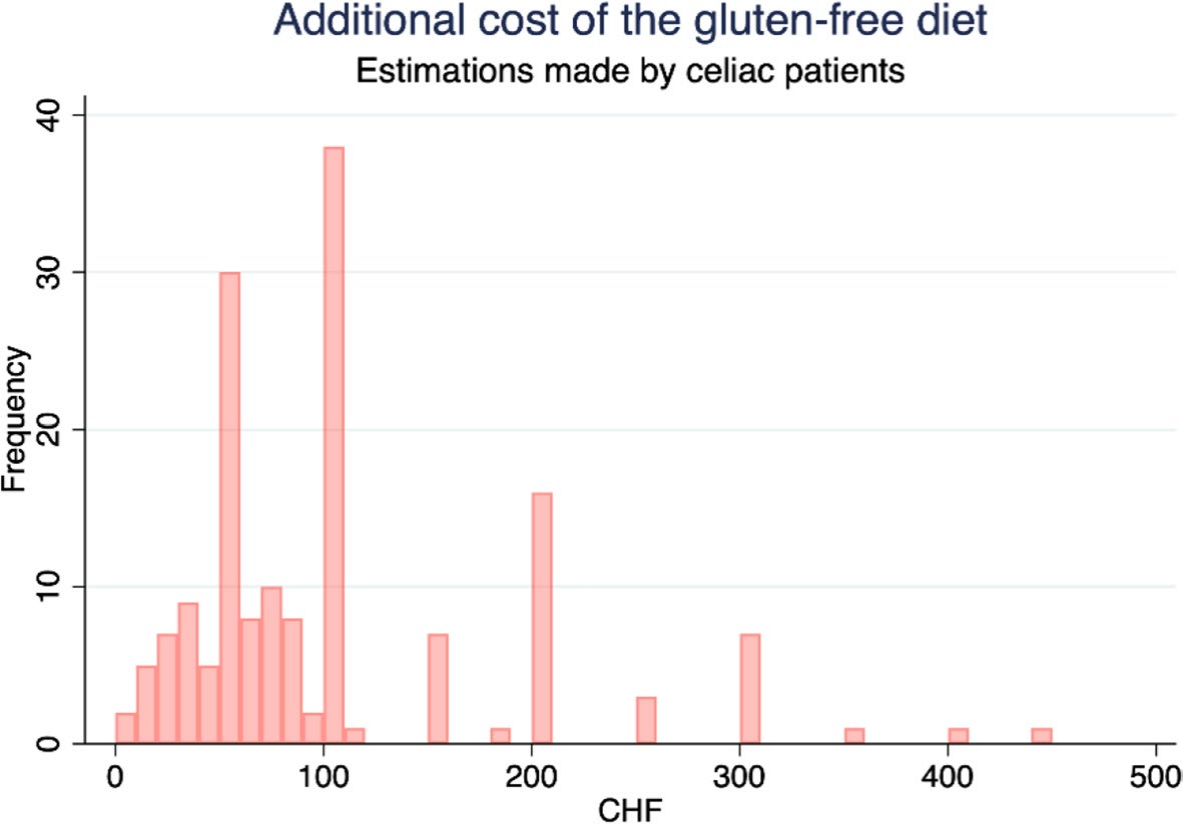
Monthly expenses due to the gluten-free diet, estimated by celiac patients

This procedure assumes that celiac patients have the same consumption pattern as non-celiac consumers. This assumption is likely to be strong, since celiac patients adjust to prices and to the availability of products, like any other economic agent. Therefore, for comparison purposes, we compare the estimates from Table 4 with patients' own estimations presented in Soler [35] and summarized in Table 5 and Fig. 3. According to celiac patients, the extra costs implied by GFD are about CHF 102 per month and per person, on average, CHF 1224 per year. This corresponds to a budget increase of 223% compared to a regular diet.

**Table 5:** Monthly expenses due to the gluten-free diet, estimated by celiac patients

Variable	Obs	Mean	Std Dev	Min	Max
Additional cost of GFD (CHF)	162	102	81.65	0	450

## Limitations and considerations for future research

This analysis confirms the previous results obtained in the literature. Some gluten-free products are up to 212% more expensive but, on average, the premium amounts to 79%. Most gluten-free products are hence more expensive than regular products but cheaper than predicted by most of the other articles reviewed in Section “Literature review”. This might be because the general price level is already quite high in Switzerland compared to other similar countries and imported gluten-free products or raw materials might not be much more expensive than regular ones in Switzerland.

Regarding the budget increase, one observes that the estimation given by respondents in the survey from Soler [35] is much larger than our present estimation based on our database of prices and the budget data from FSO. The survey responses might be overestimated since it is likely difficult to evaluate the difference in price, if one is not used to buy regular products anymore. The results, however, seem to consistently indicate a substantial higher price for gluten-free food.

We should note that our present budget analysis is limited by the assumption that people with gluten-related disorders have the same consumption pattern as the average Swiss person. This consumption could however differ for several reasons: price differences might affect choices, but most probably the lack of availability and lower palatability of given GF products might lead to a smaller consumption of these categories of products. That being said, it is hard to evaluate whether this would affect our estimation with an upwards or downward bias.

Our estimation might also be an underestimation of the true additional cost due to the type of shops considered in the study. Indeed, although our hedonic pricing analysis covers 70% of market shares, the database does not cover more than two retailers. Many people with gluten-related disorders probably buy some products on other specialised websites or shops (such as pharmacies), which are generally more expensive than supermarkets. In addition, the quality of the gluten-free food is probably not completely comparable to the regular food, implying that celiac patients probably choose the pricier options if affordable and available. This means that taking the average increase might be an underestimation of the additional cost.

Taking these arguments into consideration, the true increase in the budget for people with gluten-related disorders probably lies in between the patients' own estimation and the price-based estimate.

To be able to get a more precise estimate, further research should study the basket of food products purchased by the households with gluten-related disorders and compare it with regular Swiss households’ consumption studied by the Federal Statistical Office. This would enable to see the shift (if there is one) in consumption towards other products that do not contain gluten, and estimate the cost of the GFD more precisely.

Other research should be carried out on celiac disease in Switzerland. In particular, a study on the affordability of the gluten-free diet for the lower-income patients would be very useful and interesting. Some kind of gluten-free price index could be developed to track the variations in price over the years and in comparison to regular products.

Given the gained interest in GFD, the supply (and diversity) of gluten-free foods is probably going to grow over the next years, we expect that prices will probably adapt downwards a little, as producers realize economies of scale and of scope. However, a mark-up on the price will probably remain, as producing gluten-free food requires special certified raw materials, and the gluten-free doughs are in general more difficult to work with because of their consistency. In addition, factories must be very careful to exclude any risk of cross-contact, which is also costly, in particular if there is an accident and they have to call back the products.

## Conclusion

The medical costs for celiac disease are covered by the Swiss health insurance, as would other diseases be covered. However, Swiss public support for adult patients with celiac disease is rather low when it comes to the additional cost of gluten-free food in comparison to other countries, and as a reminder, it should be noted that GFD is the only available treatment for celiac disease. At present, for adult celiac patients, only the taxation system provides support for the additional cost of gluten-free diet under the form of a deduction on the income tax. Different possibilities to cover the cost of the diet exist in other countries, namely food provision, food prescriptions, fixed-rated transfers and tax deductions (similar to Switzerland) [17].

The introduction of a policy compensating celiac patients could modify the market equilibrium as it could influence the willingness-to-pay of celiac patients and the pricing strategy of the producers. These consequences should therefore be properly studied in the design of the policy measure, so that producers do not take advantage of it, by increasing their mark-up. In addition, measures that directly address celiac patients would probably be more cost-efficient than market measures, such as subsidies, given the propensity of non-celiac patients to follow a GFD.

Our hedonic pricing approach uses a rich database of food products provided by the two biggest food retailers in Switzerland. Disentangling the impact of products' characteristics on the price thanks to the hedonic pricing method shows that gluten-free food is indeed more expensive than regular food. This premium amounts up to 212% for some categories of products, on average to 79%. Combining our results with the Swiss households' budget survey, we find that that GFD adherent bear an extra financial burden of 421 CHF per year, an increase of 77%. While this may be a choice for certain people, those with gluten-related disorders must follow a strict GFD and incur these costs. If celiac disease and other forms of gluten-intolerances were, as they should, considered as diseases, it would make sense that the health insurance covers the extra costs of the only existing treatment, i.e. the GFD.

## Data Availability

The datasets used and/or analysed during the current study are available from the corresponding author on reasonable request.

## Appendix

**Table 6:** Budget increase using results from the linear model

**OFS Categories **	**Corresponding categories from our database **	**Monthly cost per household (2.17 people) for a regular diet**	**Annual cost per person per year for a regular diet**	**% increase for gluten-free products**	**GF annual premium**	**Annual cost for GFD per person**
5111.02: Pâtes alimentaires	Pasta	7.45	41.19	0.59	24.30	65.45
5111.03: Pain	Bread (ofs)	25.79	142.61	1.13	161.15	304.03
5111.04: Pâtisserie sucrée ou salée	Average of Muffins and biscuits	41.58	229.96	0.51	117.28	346.13
5111.06: Farine de blé	Flour (ofs)	1.12	6.21	1.28	7.95	14.15
5111.07: Autres farines, fécules, semoules, flocons et graines de céréales	Average of Wholemeal flour (ofs), Couscous, Muesli and Cereals	1.72	9.50	0.55	5.23	14.75
5111.08: Autres produits à base de céréales	Average of Crisp bread, Pizza, Dough	10.33	57.11	1.22	69,67	126.99
5213.00: Bières avec et sans alcool	Beer	10.87	60.11	0.60	36.07	96.07
**Total **		**98.86**	**546.69**	**0.77**	**421.65**	**967.58**

All amounts are in Swiss francs. Source : own database of prices and OFS [34]

**Table 7:** Results of the model with Box-Cox transformation on the dependent variable

Price 100g	Coefficient	Std. err.
/theta	0.132***	0.037
**Estimates of scale-variant parameters**	**(1)**	
	**Price per 100 g **	
Organic	0.074	
Wholemeal	0.142	
Premium	0.470	
Budget	-0.967	
Pasta	0.511	
Bread	0.714	
Toasts	0.683	
Pre-cooked bread	1.118	
Crisp bread	1.400	
Dough	1.030	
Couscous	0.682	
Biscuits	2.012	
Cereals	1.075	
Muesli	1.209	
Muffins	1.580	
Pizza	1.524	
Soy sauce	1.869	
Beer	0.464	
GF pasta	0.431	
GF bread	0.787	
GF toasts	0.854	
GF Pre-cooked bread	0.683	
GF crisp bread	0.472	
GF dough	0.873	
GF couscous	0.263	
GF biscuits	0.223	
GF cereals	0.183	
GF muesli	0.338	
GF flour	0.719	
GF Muffins	0.657	
GF Pizza	1.145	
GF soy sauce	0.129	
GF beer	0.416	
Constant (Reg. Flour)	-1.272	
sigma	0.279	
Observations	551	

Heteroskedasticity-robust standard errors reported in parenthesis. * *p* < .1, ** *p* < .05, *** *p* < .01

## Abbreviations

FSO: Federal Statistical Office
GF: Gluten-free
GFD: Gluten-free diet

## Footnotes

1 It should be noted that some celiac patients, despite suffering from the auto-immune reaction and the related negative health consequences, do not experience “visible” symptoms [36]. In this sense, eating gluten still affects their intestine and they may also experience visible symptoms in the long run.

2 Exchange rate USD-CHF was approximately 1 in 2019. All other currencies have been converted to USD using the data collection year exchange rate.

3 At that point in time, children until 20 years of age benefited from a subsidy, up to CHF 1450 per year, given by the disability insurance. This compensation was removed in 2022.

4 See for example [37].

5 To be more precise, with a first-degree Taylor approximation, (with X as an independent dummy variable, beta its estimated coefficient, and *price* as the dependent variable), we can say that: $\upbeta =\text{ln}\left(price \right|X=1)-\text{ln}\left(price \right|X=0)= \frac{\text{ln}\left(price \right|X=1)}{\text{ln}\left(price \right|X=0)} \approx \frac{price| X=1}{price | X=0}-1=\%\Delta price$. This will only be precise if we estimate small variations in price. For greater variations, it is better to calculate the real percentage change by taking: ${e}^{\beta }-1$.
